# Genetic diversity of Atlantic Bluefin tuna in the Mediterranean Sea: insights from genome-wide SNPs and microsatellites

**DOI:** 10.1186/s40709-017-0062-2

**Published:** 2017-02-16

**Authors:** Aglaia Antoniou, Panagiotis Kasapidis, Georgios Kotoulas, Constantinos C. Mylonas, Antonios Magoulas

**Affiliations:** Institute of Marine Biology, Biotechnology and Aquaculture (IMBBC), Hellenic Centre for Marine Research, Gournes Pediados, P.O. Box 2214, 71003 Heraklion, Crete, Greece

**Keywords:** Atlantic Bluefin tuna, *Thunnus thynnus*, Mediterranean Sea, Microsatellites, Genome-wide SNPs, ddRAD-seq, Genetic diversity

## Abstract

**Background:**

Elucidating the patterns of the Atlantic Bluefin tuna [ABFT, *Thunnus thynnus* (Linnaeus, 1758)] population structure constitutes a challenging task of great importance. Most of the unique challenges stem from its biology, as well as the attributes of the marine realm in which it disperses. Accurate information is urgently needed for stock assessment, and the identification of critical features to the persistence and adaptation of populations in order to formulate and adopt effective strategies for ABFT conservation and management. Conclusions of a great number of ABFT genetic studies on the Mediterranean Sea stock structure are rather controversial and not yet conclusive. In this study, ABFT genomic diversity was investigated in the Mediterranean Sea, which is the most important area for the species’ reproduction.

**Results:**

Analyzing genome-wide SNPs and microsatellites from ABFT samples collected throughout the Mediterranean Sea did not provide strong evidence of genetic structure, pointing towards the existence of a single panmictic unit. An alternative view would recognize a failure to reject the null hypothesis of a panmictic unit as an effect of the study’s sampling design, the type of markers used, and the effectiveness/suitability of analysis methods in respect to the species biological characteristics or any combination of the above.

**Conclusions:**

Unravelling the drivers of ABFT population diversity would require the consideration of important aspects of the species spawning behavior for the determination of the appropriate sampling design. Novel approaches and methods of analysis that will bring together experts in genetics/-omics, ecology and oceanography are deemed necessary. Analyzing ABFT genetic data under the discipline of seascape genetics could provide the analysis framework under which major abiotic and biotic forces controlling ABFT recruitment could be identified, elucidating the complicated population dynamics of the species, while multiple and continuous fisheries monitoring should in all cases be considered as a prerequisite in order to achieve efficient and long-term ABFT conservation.

## Background

Describing the patterns of population subdivision in continuously distributed populations has always been a challenge in population genetic studies. Some of the most important aspects regarding genetic data analysis include the model’s assumptions, which, when met, lead to insightful realizations. However, given that assumptions are tailored to certain study systems, their appropriateness is depended upon the pattern of population subdivision. As a result, they are rarely met in real studies, while basic biological processes with evolutionary implications (e.g. migration and spatial heterogeneity) are often not taken into consideration [1, 2]. Furthermore, since different population histories can lead to the same observed pattern of genetic diversity [3], the recovery of the true population genetic structure cannot be guaranteed in all cases despite the development of powerful procedures to detect population subdivision. One of the most promising future prospects include the disciplines of landscape and seascape genetics that provide a step towards the elucidation of such cases, by combining the ecology, genetics and environmental demands of the studied species. Emphasis is given to the individuals’ dispersal process focusing on understanding how movement of an organism through the landscape or seascape impacts realized dispersal and gene flow [4].

Understanding and quantifying dispersal processes in marine settings and the impact of spatial factors to genetic changes over both space and time is an extremely difficult task with most of the unique challenges stemming from the biology of marine taxa and the fluid medium in which they disperse [4, 5]. The genetic patterns observed in marine populations have been shaped by the combined result of a suite of interacting forces and traits such as demography, species’ life history traits, rates of migration influenced by spatial factors, lingering signals of history, influences of local ecology and/or local adaptation, some degree of noise and study design factors. Seascape genetics focuses on uncovering support for effects of these forces in the spatial genetic structure [4].

The Atlantic Bluefin tuna [ABFT, *Thunnus thynnus* (Linnaeus, 1758)] constitutes such a challenging case, since it represents an animal with a wide geographic distribution, high potential for dispersal and interesting life history traits (i.e. spawning fidelity). These attributes necessitate the employment of methodologies that could provide more accurate information needed for stock assessment, and the identification of critical features to the persistence and adaptation of populations, based on those effective strategies for its conservation and resource management that could be formulated [6]. Knowledge on species biological traits and critical parameters that influence its viability is of a great importance given that ABFT is highly exploited, with its fisheries having experienced substantial declines for many years [7–11] being listed as Near Threatened in the European marine region (Regional assessment) by the International Union for Conservation of Nature Red List (IUCN). For management purposes, the International Commission for the Conservation of Atlantic Tunas (ICCAT) considers the existence of two separate Atlantic stocks with very little mixing among them: the eastern (that includes the Mediterranean) and the western stock with IUCN stock status being assigned to over-exploited and depleted, respectively. According to the latest ICCAT Report for the Biennial Period 2014–2015 [12], most of the updated fisheries indicators are concordant with a more optimistic perception of status for both species stocks that need however to be further confirmed by future data and analyses. The Mediterranean Sea is an important area for the ABFT reproduction, hosting all known spawning sites for the eastern stock [13–18]. Although there is a great number of studies suggestive of an existing stock structure within the Mediterranean, both in terms of physiology and behavior i.e. philopatry and natal homing [11, 16, 18, and references therein], the conclusions based on genetic studies are rather controversial and not yet conclusive [11, 19–22]. Many of those studies provide evidence of a two-unit structure within the Mediterranean Sea (western and eastern stock), while in the study of Riccioni et al. [22] there are strong indications of a population structure that is not on the west-east axis, but depends on environmental factors such as salinity and mean surface temperature.

These rather conflicting results could be due to several sources rendering standardization of ABFT genetic analyses extremely important for the study of the species population structure [23]. In most ABFT studies, as in other marine organisms, sampling is primarily population-based where many individuals are being collected from each sampling locality with typically <15 locations being studied in total. This combined with the geographic scale over which marine organisms are likely to disperse and the spatio-temporal scales of seascape features make sampling hundreds of individuals evenly (or at random intervals) along thousands of kilometers logistically challenging [5], and raises sampling design to a potential source of discrepancy between studies. Further sources may include the type of markers used, since, based on their attributes, different aspects of an organisms’ evolutionary history can be illuminated. Moreover, the methodology being employed when analyzing the data is crucial and need to appropriately take into account the type of marker(s) being used, the organisms’ life history traits and the questions addressed [24].

In the present study, both genetic and genomic methods were employed for the first time in ABFT, analyzing samples from throughout the Mediterranean Sea as well as from the Moroccan coast in the Atlantic Ocean in terms of microsatellites and genome-wide SNPs sampled by double-digest Restriction Associated DNA sequencing (ddRAD-seq). Our main focus was on selectively neutral processes by the identification of natural barriers and the estimation of levels of gene flow. In order to accomplish our goals, various statistical approaches were employed i.e., methods that apply on different models both spatial and non-spatial as well as non-model based methods, in an attempt to discriminate the direct and/or indirect key factors (i.e. seascape features and/or environmental conditions) that might have shaped the observed genetic diversity and to shed light on some of the unknown biological aspects of the species. Neutral genetic markers were selected as they are considered ideal for inferring demographic processes, such as isolation or migration among populations, given that strong selection can alter allele frequencies for selected loci relatively rapidly, and thus obscuring historical patterns.

## Methods

### Sample acquisition and DNA extraction

**Fig. 1:**
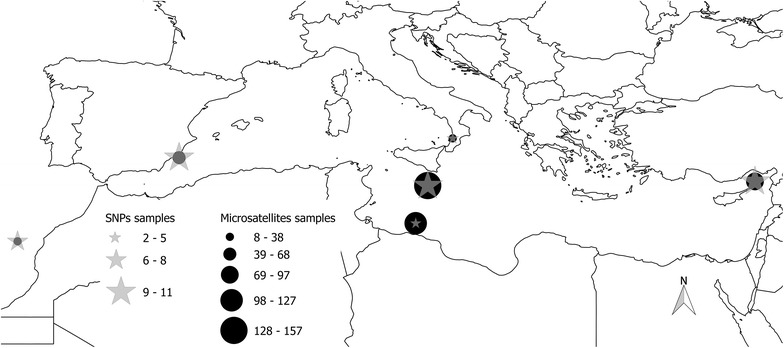
Sampling sites of ABFT. *Symbols* indicate the geographic origin of samples genotyped for microsatellite loci (*black circles*) and SNPs (*grey stars*) with size proportional to the number of analyzed samples

Tissue samples of adult ABFT were obtained from different sites throughout the Mediterranean Sea in the framework of the SELFDOTT project (EU Seventh Framework Programme, GA 212797, https://sites.google.com/site/selfdottpublic/news). Specimens have originated from Spain (broodstock from farming facilities at El Gorguel, Cartagena, southeast Spain), initially caught in the Balearic Sea (June 2007), from Malta (broodstock from farming facilities at Marsaxlokk Bay) initially caught in the waters south of Malta (June 2008 and 2009) and from Italy (project ALLOTUNA PS-085 EU Strategic funds) from farming facilities based off the coast of Vibo Marina in Calabria, southern Italy, initially caught by purse seine nets from the spawning grounds around the Aeolian Islands in the south Tyrrhenian Sea (May and June 2007). Samples from the eastern Mediterranean spawning grounds (off the coasts of Syria, January 2010), and central Mediterranean Sea (south of Malta) were also obtained from commercial ABFT fattening operations, at the time of harvesting the fish [Bluefin Tuna Hellas S.A. (Greece), Tuna Grasso S.A. (Spain), Malta Fish Farming S.A. (Malta)]. Furthermore, 14 samples were obtained from the eastern Atlantic Ocean (off the coast of Morocco). In total 67 samples from the western, 265 from the central and 96 from the eastern Mediterranean Sea were analyzed together with 14 from the eastern Atlantic, covering the majority of the species’ spawning areas within the Mediterranean Sea (Fig. 1). For a total of 442 tissue samples, total genomic DNA extraction was carried out based on a standard proteinase K protocol [25].

### Microsatellite loci genotyping

All samples were genotyped for 16 microsatellite loci: *Tth12*-*29*, *Tth185*, *Tth204*, *Tth207*, *Tth1*-*31*, *Tth16*-*2*, *Tth217*, *Tth226*, *Tth157*, *Tth4*, *Tth14*, *Tth208*, *Tth62* [26], and *Tth8*, *Tth34*, *Tth38* [27]. Microsatellite loci were optimized and combined in 4 multiplexed schemes (Mpx1: *Tth12*-*29*, *Tth185*, *Tth204*, *Tth207*, *Tth8*, Mpx2: *Tth1*-*31*, *Tth16*-*2*, *Tth217*, *Tth226*, *Tth38*, Mpx3: *Tth157*, *Tth4*, *Tth34*, Mpx4: *Tth14*, *Tth208*, *Tth62*). PCR amplification conditions consisted of: 1.5 mM MgCl_2_, 0.15 mM dNTPs, 0.125 μM of each primmer, 0.5 U Taq (Gennaxon, Ulm, Germany) in a total volume of 20 μl. Reactions were carried out using an initial step at 94 °C for 2 min, followed by 35 cycles of denaturation at 94 °C for 60 s, annealing at different temperatures depending on the multiplex PCR scheme (Mpx1 at 60 °C, Mpx2 at 54 °C, Mpx3 and Mpx4 at 57 °C) for 60 s and extension at 72 °C for 60 s, and a final extension at 72 °C for 10 min. Alleles were resolved by electrophoresis on an ABI Prism 3700 DNA Analyzer (Applied Biosystems, CA, USA). Genotypes were determined using the software STRand (http://www.vgl.ucdavis.edu/STRand). In order to minimize microsatellite alleles miscalling, the binning of alleles was accomplished using FLEXIBIN 2 [28] the output of which was manually evaluated.

We used MICROCHECKER v. 2.2.3 [29] on the complete Bluefin tuna microsatellite dataset (i.e. all 16 loci), to test for genotyping errors due to stuttering, allelic drop out and the presence of null alleles. Based on the results, three loci were excluded from further analyses (i.e. *Tth207*, *Tth208*, and *Tth38*). Furthermore, samples that yielded genotypes for less than nine loci were also excluded from further analyses providing a final dataset of 428 samples representative of all studied sites.

### Genome-wide SNPs collected by ddRAD-seq

Forty-eight ABFT samples yielded the required DNA both in terms of quality and quantity, enabling their use in the next generation sequencing protocol. Those samples were representative of all studied sites (i.e. Eastern Mediterranean Sea: 12 samples, Central Mediterranean Sea: 17 samples—6 from Italy and 9 from Malta M. Bay and 2 from south of Malta—, Western Mediterranean Sea: 12 samples, off Morocco coasts: 7 samples, Fig. 1). DdRAD-seq data were collected following the protocol described by Peterson et al. [30]. We double-digested 400 ng of each samples’ genomic DNA using *Sbf*I (restriction site 5′-CCTGCAGG-3′) as a rare cutter and *Msp*I (restriction site 5′-CCGG-3′) as a common cutter in a single reaction in accordance to the guidelines of the manufacturer (New England Biolabs, MA, USA). Fragments were purified with Agencourt AMPure XP beads (Beckman Coulter, IN, USA) in all steps of the library preparation. The oligonucleotide sequences used for barcoding and Illumina indexes during library preparation are provided in Peterson et al. [30]. The libraries were size-selected (between 415 and 515 bp including the length of the adaptors) on a Pippin Prep size fractionator (Sage Science, MA, USA). The final library amplification used proofreading Taq and Illumina’s indexed primers. The fragment size distribution and concentration of each pool were determined on an Agilent 2100 Bioanalyzer (Agilent, CA, USA), and qPCR was performed to determine the concentration of the sequencing target fragments of each library before multiplexing equimolar amounts of each pool for sequencing on a half Illumina HiSeq2500 lane (100-bp, single-end reads) at the STAB Vida facility (Caparica, Portugal).

Raw Illumina reads were processed using the program pyRAD v 3.0.5 [31]. Samples were demultiplexed using their unique barcode and adaptor sequences. Sites with Phred quality scores under 99% (Phred score = 20) where changed into “N” characters, and reads with ≥4% N’s were discarded. Each locus was reduced from 100 to 89 bp after the removal of the 6-bp restriction site overhang and the 5-bp barcode. The filtered reads for each sample were clustered using the program VSEARCH v.1.1.3 (https://github.com/torognes/vsearch) and MUSCLE v.3.8.31 [32], establishing homology among reads within samples. The assembly of the ddRAD-seq data was performed using 95% as a clustering threshold given the intra specific nature of our dataset. Consensus sequences that had a low coverage (<6 reads), excessive undetermined or heterozygous sites (>4), or too many haplotypes (>2 for diploids) were discarded. The consensus sequences were clustered across samples using the same threshold used to cluster data within each sample (i.e. 95%). Each locus was aligned with MUSCLE v.3.8.31 [32] and a filter was used to exclude potential paralogs i.e. loci with excessive shared heterozygosity among samples. A relaxed filter allowing a maximum of three samples to be heterozygous at a given site (paralog = 3) was also applied.

Samples with low loci recovery were removed from the dataset (four samples in total i.e. one sample from Italy, one from Spain, one from Morocco and one from Syria). This step allowed the inclusion of 44 (out of 48) samples in our SNPs dataset for further analyses.

The final ddRAD-seq loci were assembled by adjusting the minimum individual value (min. ind.: specifying the minimum number of individuals that are required to have data present at a locus in order for that locus to be included in the final matrix), (1) to 40 (allowing maximum 4 samples to have missing data for each locus that is 10% missing data, SNPs dataset 1), and (2) to 44 where missing data were not allowed (0%, SNPs dataset 2).

### Data analysis

The ABFT specimens were grouped into six predefined populations based on their geographic origin, namely, one from eastern Mediterranean Sea (off the coasts of Syria), three from central Mediterranean Sea (two in Malta—Malta M. Bay, Malta South—and Italy—Vibo—), one from western Mediterranean Sea (Spain) and one from Morocco (Fig. 1). Hardy–Weinberg equilibrium was evaluated for all loci using GENEPOP on the Web [33] (http://genepop.curtin.edu.au/). Comparative measures of genetic diversity and the F_ST_ index, assessed by the estimator θ [34], employed as a measure of genetic differentiation and the level of gene flow among the different geographic locations within the Mediterranean Sea, were estimated using GENETIX v 4.05 [35].

In an attempt to acquire insight into the demographic and evolutionary processes that have shaped the genetic patterns of ABFT in the Mediterranean Sea, for both types of datasets (microsatellites and SNPs), three statistical methods were employed exhibiting different strengths and limitations. Those methods cover a wide range of levels of population structure and patterns of genetic diversity generated by different evolutionary processes i.e. two Bayesian clustering methods, principal-component analysis (PCA) and a method for the estimation of effective migration surfaces (EEMS). Clustering methods are better suited in cases with a medium to strong signal of population structure (i.e. the presence of genetically distinct groups), where sampling localities may or may not be in use, where admixture events are recent and there is no isolation by distance. PCA are multivariate descriptive methods that unlike Bayesian clustering methods, do not rely on explicit population genetics models, and they are preferable when many loci are available and the structure is subtle [36, 37]. The PCA methods can generally handle and diagnose patterns of isolation by distance [38, 39]; however, they are influenced by sampling biases [40–42] something that might be the case not only in our dataset but also in other already published ABFT datasets, and ignore sampling locations even if they are known. Estimation of effective migration surfaces is the third method that was employed, which displays population structure from geo-referenced genetic samples when it is broadly and perhaps not entirely consistent with isolation by distance. It produces a visual representation of spatial patterns in genetic variation and highlights regions of higher-than-average and lower-than-average historical gene flow, and as such can identify potential corridors and barriers to gene flow. Estimation of effective migration surfaces is specifically applicable when there is not strong population structure and where genetic similarity tends to decay with geographic distance but where this decay with distance may occur more quickly in some regions than in others (i.e. the data conform roughly to isolation by distance). In comparison to PCA methods, EEMS is better suited to discern migration scenarios and is less sensitive to the underlying sampling scheme. The EEMS also estimates the effective diversity rate within each deme reflecting the expected genetic dissimilarity of two individuals sampled from one location [43].

Patterns of population structure were investigated using two Bayesian clustering approaches implemented in STRUCTURE v. 2.3.4 [44] and GENELAND v. 4.0.5 [45]. STRUCTURE analysis was employed as a non-spatial clustering method where the assumed prior for the clustering is uniform and therefore all clustering solutions are equally likely. We used a burn-in period of 200,000 and 800,000 MCMC steps for different values of K ranging from 1 to 10. Using longer MCMC runs did not modify the results. We used the admixture model, where each individual is assumed to have inherited some proportion of its ancestry from each population. In this model, individuals are clustered jointly into two or more populations if their genotypes indicate that they are admixed. The correlated allele frequency model (F-model) was employed. This model corresponds to a demographic scenario of simultaneous divergence of subpopulations from an ancestral population, with each subpopulation undergoing genetic drift in allele frequencies at a unique rate inversely proportional to its effective size [46], allowing at the same time individuals of mixed ancestry [44]. We did not use a priori information about population affiliation. Each run (for a fixed K) was repeated 5 times in order to check the stability of the results.

GENELAND was employed as a better definition of spatial genetic units by integrating spatial coordinates of samples. An explicit model is employed which describes the fact that differentiated populations tend to be spatially structured occupying distinct areas, and maps of population ranges are being generated. It incorporates a non-admixture model assuming that each individual originates purely from one of the defined genetic clusters [47]. Moreover, it is a fully Bayesian approach, in the sense that the number of populations is treated as a parameter processed by the Markov Chain Monte Carlo (MCMC) scheme without any approximation [48]. We used 10^6^ iterations for each run, including a burn-in of 10,000 iterations, and a sampling frequency of 1000.

The PCA analysis was performed with the R (v. 3.2.5, [49]) package ADEGENET v. 2.0.0 [50]. In the analyses of all different datasets, allelic frequencies were scaled using the function scaleGen and replacing missing data with the allele means and scale frequencies.

The EEMS analysis was run for all generated datasets (microsatellite and both SNPs datasets, i.e. 10 and 0% missing data). Furthermore, in order to detect any bias that might be due to the present study’s sampling scheme, EEMS analysis was employed to the seven microsatellite loci dataset of Riccioni et al. [22] which can be considered as complementary—in respect of sampling—exhibiting population structure associated with environmental factors (i.e., with high information content). All EEMS analyses were performed with three different grids i.e. 200, 300 and 500 demes. Preliminary runs were made in order to define and fine-tune acceptance ratios to reach an optimal 20–30% for most of the parameters. Fine-tuning was performed by modifying the proposal variances as follows: mSeedsProposalS2 = 1.5 for microsatellite datasets and 2.0 for SNP datasets, and qSeedsProposalS2 = 1.5, mEffctProposalS2 = 5.5, qEffctProposalS2 = 0.05, mrateMuProposalS2 = 0.5 for all datasets. For each grid we performed five replicate analyses, each with a different random seed, in order to assess convergence of the chain. All EEMS analyses were run for 10^7^ iterations, with a burn-in of 10^6^. Results were averaged across all the independent realizations.

## Results

### Microsatellites

Deviation from Hardy–Weinberg (HW) equilibrium was detected (highly significant probability test) when considering ABFT individuals as a single population. Four loci (*Tth16*-*2*, *Tth226*, *Tth4* and *Tth8*) displayed heterozygote deficit, and two (*Tth14* and *Tth34*) excess of heterozygosity, when α = 0.05. All loci exhibited a high number of alleles ranging from eight (*Tth157*) to 30 (*Tth4*).

Measures of genetic diversity of the six predefined populations are depicted in Table 1. The mean number of alleles and the levels of heterozygosity are of the same magnitude between the geographic areas within the Mediterranean Sea in respect of sampling size. Private alleles were detected in all studied areas except Italy, which is probably due to the low number of studied samples. The F_ST_ values are depicted in Table 2, with ten out of fifteen being statistically different from 0 ranging from 0.00175 (Malta M. Bay–Syria) to 0.012 (South Malta–Morocco). It is worth noticing that F_ST_ values associated with the Italian predefined population did not statistically differ from zero. Furthermore, F_ST_ values concerning all the rest geographic areas are statistically differentiated (i.e. low F_ST_ values that significantly differ from zero).

**Table 1:** Measures of microsatellite genetic diversity in the predefined ABFT populations

Predefined populations	Number of samples	Average number of alleles/locus	H_o_	H_e (n.b.)_	HW (*p* value)	Het. deficit	Het. excess
Syria	92	11.46	0.7938	0.7732	0.2419	2/13	3/13
Malta M Bay	151	13.38	0.7774	0.7685	0.0001	3/13	2/13
South Malta	99	12	0.8181	0.7685	High. Sign.	0/13	3/13
Italy	7	5	0.7408	0.7127	0.6838	0/13	0/13
Spain	66	12.23	0.7449	0.7537	0.5800	0/13	2/13
Morocco	13	7.54	0.7908	0.7903	0.1186	2/13	1/13

H_o_: observed heterozygosity, H_e (n.b.)_: unbiased expected heterozygosity

**Table 2:** Estimated F_ST_ values between the predefined ABFT populations using 13 microsatellite loci

F_ST_	Syria	Malta M Bay	South Malta	Italy	Spain	Morocco
Syria	–	0.00175**	0.00188**	0.00452	0.00356**	0.01085**
Malta M Bay		–	0.00249**	0.00691	0.00234**	0.00813**
South Malta			–	0.00771	0.00376**	0.01240**
Italy				–	0.00627	0.02058
Spain					–	0.00859**
Morocco						–

Significance of values was assessed with 20,000 permutations and significant values are indicated with ** (α = 0.05)

The clustering analysis of STRUCTURE based on the admixture model with no use of the sampling locations of individuals, could not be performed given that the estimate of α (alpha parameter reflecting the degree of admixture) varied greatly throughout the run (i.e. >0.2). The problem was not fixed even after the increase of ALPHPROPSD parameter that was performed in an attempt to improve mixing (as suggested by Pritchard et al. [51]). Such a behavior could be due either to departures from the model assumptions or lack of signal in the data. No signal of population structure was indicated by GENELAND analysis with the most probable number of clusters being K = 1.

**Fig. 2:**
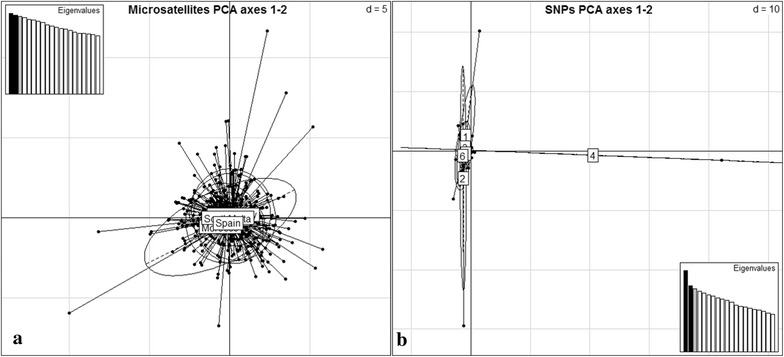
Principal component analysis (PCA) plots of ABFT samples employing **a** 13 microsatellite loci and **b** 441 SNPs from ddRAD-seq (1: Syria, 2: Italy, 3: Malta M. Bay, 4: South Malta, 5: Spain, 6: Morocco). *Black circles* represent genotypes and inertia ellipses ABFT predefined populations

 The PCA analysis indicated that the genetic similarity among the predefined populations of ABFT at 13 microsatellite loci was high and did not reveal any population structure reflecting the geographic origin of the samples. The results of the analysis are presented graphically along the first and second axes in line with eigenvalues in Fig. 2a. This coincides with the results obtained by model-based analyses employed in this study (STRUCTURE and GENELAND). The eigenvalues of each of the first two axes were not exceeding 1.46% (PC1: 1.493%, PC2: 1.459%).

**Fig. 3:**
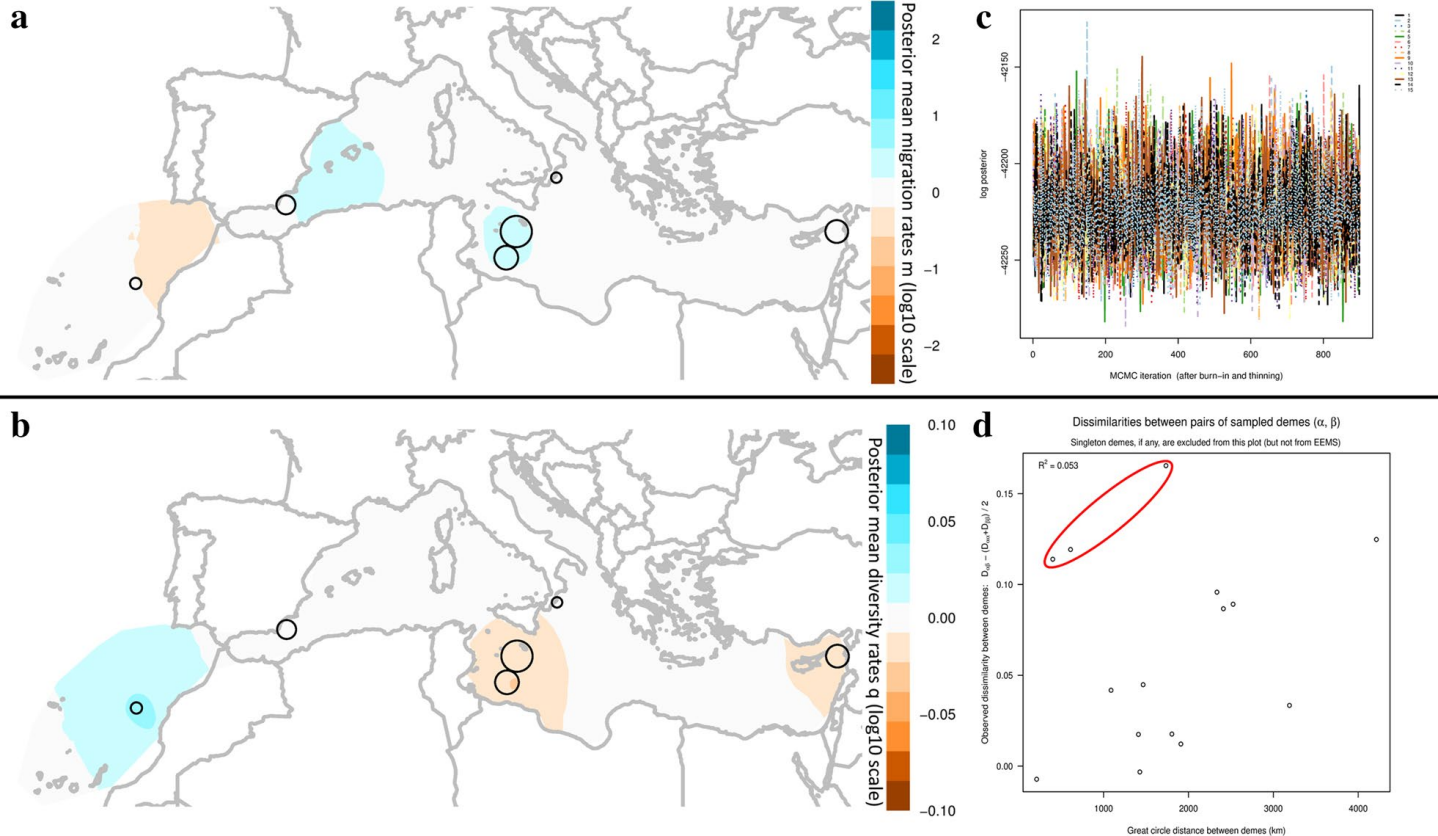
EEMS analysis of 428 samples of Bluefin tuna from the Mediterranean and off Morocco coasts based on 13 microsatellite loci. *Empty circles* correspond to the studied geographic sites with their sizes being proportional to the number of samples analyzed in the present study. **a**, **b** Averaged, over different grids, effective migration rates (m), and diversity contour plots (q) respectively, **c** diagnostic plot of MCMC iterations vs log posterior of all runs (n = 15) where there is no indication of non-convergence, **d** *scatterplot* of the observed between demes pairwise genetic differences against the geographic distances of the demes (Great circle distances in Km). The coefficient of determination is indicated at the* top left* of the scatterplot (R^2^ = 0.053). Outliers of the analysis are depicted with a red ellipse and describe genetic dissimilarities related to Vibo samples (Italy)

Regarding the EEMS analysis, there was no indication of non-convergence during any of the runs that were performed (based on the log posterior fluctuations along MCMC iterations after burn-in and thinning, Fig. 3). Furthermore, for the majority of the parameters, the acceptance ratios were near 20–30%, which is also an indication of good performance. The averaged, over different grids, effective migration and diversity contour plots across the Mediterranean estimated by EEMS are depicted in Fig. 3. In respect to the effective migration plots, although there are areas presenting slightly higher (i.e. Balearic Sea and Malta’s surrounding area) or slightly lower (Strait of Gibraltar) migration rates (Fig. 3a), those are not statistically supported (i.e. posterior probabilities <0.90). The results indicate uniform migration rates and no deviations from exact isolation by distance. Furthermore, higher effective diversity was observed in the area off Morocco coasts and lower in the surrounding areas of Malta and in the Levantine (Fig. 3b). Again, those differences were not statistically supported (posterior probabilities <0.90). The diagnostic scatterplots of between demes pairwise genetic differences are indicative of a not a good fit of the EEMS model to the data with a coefficient of determination equal to R^2^ = 0.248. On the contrary, within demes differences are better predicted with R^2^ = 0.82 (data not shown). When plotting the observed between demes dissimilarities in respect to their great circle distances, the bad fit of the model is also evident, where the coefficient of determination is equal to R^2^ = 0.053 (Fig. 3d). This is mostly due to the outliers (depicted with a red ellipse in Fig. 3d) that describe genetic dissimilarities related to the Italian samples. Removing those and running again the analysis provided identical contour maps of both migration and diversity rates while it significantly improved the fit of the model to the data. The observed vs fitted dissimilarities coefficient of determination between demes increased to R^2^ = 0.942, the within demes was equal to R^2^ = 0.935, while the coefficient of determination of the observed between demes genetic dissimilarities vs the geographic distances was also greatly improved reaching R^2^ = 0.571.

**Fig. 4:**
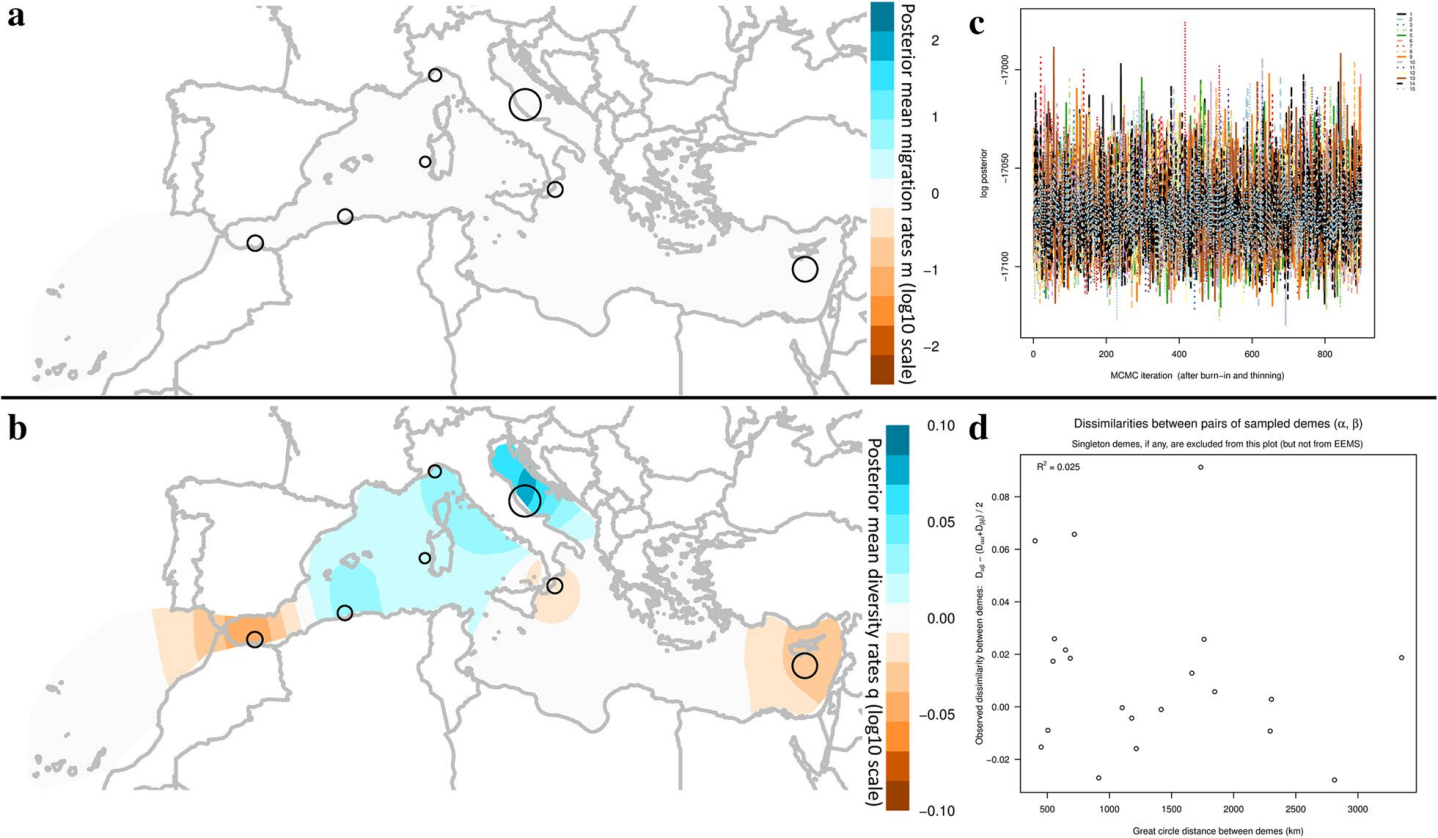
EEMS analysis of the microsatellite dataset of Riccioni et al. [22] with 316 samples of Bluefin tuna from the Mediterranean based on 7 microsatellite loci. *Empty circles* correspond to the studied geographic sites with their sizes being proportional to the number of samples analyzed in the study of Riccioni et al. [22]. **a**, **b** averaged, over different grids, effective migration rates (m), and diversity contour plots (q) respectively, **c** diagnostic plot of MCMC iterations vs log posterior of all runs (n = 15) where there is no indication of non-convergence, **d** *scatterplot* of the observed between demes pairwise genetic differences against the geographic distances of the demes (*Great circle* distances in Km). The coefficient of determination indicated at the* top left* of the scatterplot is equal to R^2^ = 0.025

In the analysis of Riccioni et al. [22] dataset and despite the fact that it exhibits spatial population structure [i.e. three clusters with distinct geographical distribution (latitudinal gradient): southern Mediterranean Sea, northern Mediterranean Sea and Sardinia] we were not able to detect barriers to gene flow. According to the EEMS results, migration rates are uniform throughout the Mediterranean Sea, and genetic diversity rates are higher for the Adriatic Sea, the Lingurian Sea, Sardinia and Algeria and lower for the Levantive, the Tyrrhenian Sea and the Alboran Sea (Fig. 4). Genetic diversity is statistically supported only for the high rates of Adriatic Sea and the low rates of Alboran Sea (posterior probabilities >0.90). Plots of the observed vs fitted differences between demes, are indicative of the poor fit of the model to the data with the coefficient of determination being equal to R^2^ = 0.054. The within demes coefficient of determination is high R^2^ = 0.986, while when plotting the observed between demes dissimilarities in respect to the great circle distances between demes, the coefficient of determination is again low and equal to R^2^ = 0.025 (Fig. 4d).

### DdRAD-seq data and SNPs

Illumina sequencing of a single read ddRADTag library from 48 ABFT samples yielded an average of 885,827 reads per sample and 42,519,712 100 bp reads in total, with a mean coverage of 30×. After quality filtering and paralog removal, 830 anonymous loci were recovered on average per sample. The number of homologous loci for at least 40 (out of the 44 samples, i.e. 10% missing data) was 856. Of these 856 loci, almost half of them (415 loci) did not contain any variable site, 262 contained one SNP, 119 loci two SNPs, 39 loci three SNPs, 15 loci four SNPs and 6 loci contained from five to eight SNPs. The total number of variable sites was equal to 714, while the sampled unlinked SNPs were 441 (SNPs dataset 1).

The number of homologous loci for all samples (0% missing data) was 336. Of these, 179 loci did not contain any variable site, 119 contained one SNP, 51 loci two SNPs, 10 loci three SNPs and 7 loci contained from four to seven SNPs. The total number of variable sites was equal to 283, while the sampled unlinked SNPs were 187 (SNPs dataset 2).

Levels of observed heterozygosity are of the same magnitude between the geographic areas within the Mediterranean ranging from 0.0250 (Malta M. Bay) to 0.0398 (Italy). Similar values were observed for SNPs dataset 2 ranging from 0.0221 (South Malta) to 0.0396 (Malta M. Bay). Hardy–Weinberg equilibrium tests and F_ST_ parameter estimates could not be performed or trusted due to the insufficient information contained in the data to compute estimates and/or confidence intervals (e.g. not enough alleles in the sample, [52]).

The clustering analysis of both STRUCTURE and GENELAND indicated no signal of population structure with the most probable number of clusters being K = 1.

The PCA analysis implied that the genetic similarity among the predefined populations of ABFT at 441 unlinked SNPs was high and did not reveal any population structuring, coinciding with microsatellite data results of this study. A two-dimensional plot based on the top two PCs is shown in Fig. 2b. This was also true for the SNPs dataset 2 with 0% missing data (results not shown). The main feature of the PCA plots of both SNP datasets is their unstructured form and the presence of few ‘outlier samples’ originating from several sampling sites. Removal of those samples had as a result the emergence of few other ‘outlier samples’ again without a specific geographic origin. The eigenvalues of each of the first two axes were not exceeding 4.85% (PC1: 4.845%, PC2: 3.942%).

**Fig. 5:**
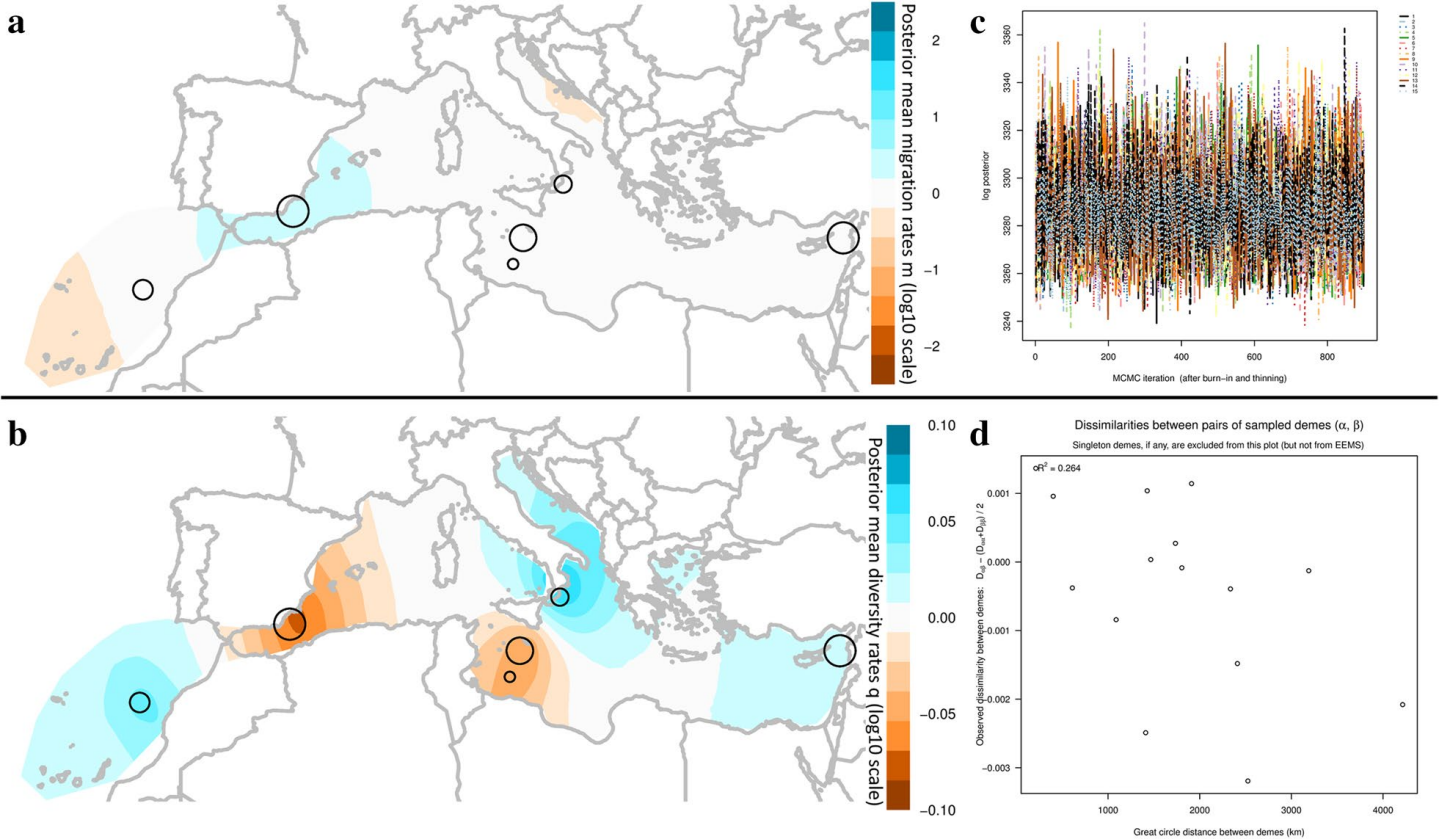
EEMS analysis of 44 samples of Bluefin tuna from the Mediterranean and off Morocco coasts based on 441 SNPs from ddRAD-seq (i.e. SNPs dataset 1). *Empty circles* correspond to the studied geographic sites with their sizes being proportional to the number of samples analyzed in the present study. **a**, **b** Averaged, over different grids, effective migration rates (m), and diversity contour plots (q) respectively, **c** diagnostic plot of MCMC iterations vs log posterior of all runs (n = 15) where there is no indication of non-convergence, **d** scatterplot of the observed between demes pairwise genetic differences against the geographic distances of the demes (*Great circle* distances in Km). The coefficient of determination is indicated at the top left of the scatterplot (R^2^ = 0.264)

The averaged, over different grids, effective migration and diversity contour plots across the Mediterranean estimated by EEMS based on 441 SNPs (SNPs dataset 1) are depicted in Fig. 5. The plot of EEMS log posterior vs MCMC iterations provides no indication of non-convergence during any of the runs that were performed on either SNPs dataset (Fig. 5c). Furthermore, for the majority of the parameters, the acceptance ratios were near 20–30%, which is also an indication of good performance.

In respect to the effective migration plots, ABFT migration in the Mediterranean is estimated to be uniform except in the area of the Alboran and the Balearic Seas where it is slightly higher. On the contrary, it is slightly lower in the South Adriatic and in the Canary Islands. However, none of the deviations is statistically significant (posterior probabilities <0.90) indicating no deviations from exact isolation by distance.

Higher effective diversity than that estimated under the model of isolation by distance is inferred for the area off Morocco coasts, South Tyrrhenian Sea and Levantine, while lower for Alboran and Balearic Seas and the area south of Malta. The higher diversity off Morocco coasts and the lower genetic diversity of Alboran Sea and South of Malta area, are statistically supported with posterior probabilities ≥0.90. The diagnostic scatterplots of between demes pairwise genetic differences predicted by the model against the pairwise genetic differences observed in the data indicate that the EEMS model is not a good fit to the data with a coefficient of determination equal to R^2^ = 0.298. The within demes differences are better predicted with R^2^ = 0.995 (data not shown). When plotting the observed between demes dissimilarities in respect to their great circle distances, the coefficient of determination is equal to R^2^ = 0.264 (Fig. 5d), also indicative of not a good fit of the model to the data.

The EEMS run for the SNPs dataset 2 (0% missing data) yielded similar results (not shown) indicating uniform migration rates that were slightly higher only at the Alboran and Balearic Seas though without any statistical support. Furthermore, the genetic diversity was higher at the areas off Morocco coasts, south Tyrrhenian, and near the Libyan coasts, while it was lower at the Alboran Sea, Malta and the Levantive with only the last one being statistically supported (posterior probability ≥ 0.95). The diagnostic scatterplots of between demes pairwise genetic differences indicate that the EEMS model is not a good fit to the data with a coefficient of determination equal to R^2^ = 0.014 while within demes differences are better predicted with R^2^ = 0.982. The coefficient of determination is equal to R^2^ = 0.005 when plotting the observed between demes dissimilarities in respect to the great circle distances.

## Discussion

Analysis of genome-wide SNPs and microsatellites of ABFT samples from throughout the Mediterranean Sea did not provide strong evidence of genetic structure, pointing towards the existence of a single panmictic unit. Microsatellite genetic diversity was high with most F_ST_ values being statistically different from zero, except those associated to the Italian ABFT specimens, a fact that could be attributed to the low number of studied samples from that area. The SNPs were characterized by the absence of population structure and low levels of heterozygosity, coinciding with the lowest SNP heterozygosities observed for the species and its congenerics [53]. These results were not anticipated given the volume of data generated in this study and the species’ life history traits, a fact that could be due to the ABFT complex population dynamics, an important aspect that needs to be elucidated.

There is a growing evidence of the complex dynamics of ABFT in the Mediterranean Sea. The ABFT, as well as many other marine fishes, such as cod (*Gadus morhua*), Atlantic herring (*Clupea harengus harengus*), and pollock (*Pollachius virens*), are characterized by spawning aggregations that occur regularly in the same geographic area every year (e.g., [54–56]). A question of interest is whether these spawning aggregations represent discrete stocks. Electronic tagging experiments of ABFT indicate extensive residency within the Mediterranean Sea by multiple year classes and a possibility of a size-dependent migration into the northeastern Atlantic [14, 17, 57]. Moreover, there are areas within the Mediterranean that seem to be isolated during the spawning season since a crossover between them has never been detected (e.g. a crossover of fish from the western Mediterranean or even the Adriatic Sea to the eastern Mediterranean basin or the opposite [16, 17]). Therefore, the existence of multiple demographic units of ABFT mixing in the Mediterranean with distinct behaviors i.e., some migratory individuals exhibiting spawning fidelity, co-existing with some resident individuals, is highly probable [17 and references therein]. Furthermore, the concepts of ‘density-dependent habitat selection’ or of a metapopulation might be more appropriate to describe ABFT dynamics, both during and outside the spawning season, than the ‘traditional’ stock concept [23]. Therefore, ABFT might be structured in multiple demographic units with their spatial-ranges in response to both environmental and fishing variability. Alternatively, ABFT in the Mediterranean Sea could be seen as a collection of discrete local populations, occupying distinct habitats, displaying their own dynamics, but with a degree of demographic influence from other local populations through dispersal [23].

In the present study, analysis of neutrally evolving markers (microsatellites and genome-wide SNPs) indicated that the studied ABFT specimens could constitute a single panmictic population that assorts randomly to spawn in different areas within the Mediterranean Sea. Microsatellite allelic richness was high throughout the study area, a fact that could be indicative of the population’s long-term potential for adaptability and persistence, but also of the importance of the Mediterranean Sea for the species. An alternative view would recognize a failure to reject the null hypothesis (under which Mediterranean ABFT constitutes a panmictic unit) as an effect of the study’s sampling design (i.e., sampling size, age categories of fish, number of sampling locations and their distances), the type of markers used (i.e. low information content), and the effectiveness/suitability of the analysis methods in respect to the species’ biological characteristics or any combination thereof. Deciding which is the case and identifying the ‘real’ causes is crucial for ABFT sustainability and conservation.

When investigating the population structure of a particular species, the limitations of the given study should always be considered. Furthermore, identification of the population structure is not always straightforward and there are cases where detection of genetic heterogeneity fails, despite its presence [58].

Given that ABFT in the Mediterranean regulates under panmixia, one might assume that the depletion of one local unit/population would be offset by the regular immigration or ongoing larval recruitment from another. However, this has not always been the case for ABFT indicating the existence of a population structure [59]. Besides that, in stocks that have been depleted by overharvest in the recent past, as the ABFT in the Mediterranean Sea, genetic data alone are not sufficient to describe some parameters of interest, for example demonstrate a high enough migration rate needed in order for the stock to be rebuilt quickly [60].

To date, the genetic studies demonstrating population structuring of ABFT in the Mediterranean basin used both temporal and spatial sampling and/or more than one genetic marker [19–21, 61]. However, in the study of Riccioni et al. [22], a pattern of genetic structuring was evident with the use of only seven microsatellite markers under an extensive sampling scheme that probably had a substantial impact on the discriminating power of their dataset.

An important aspect when analyzing microsatellite loci in fish with large populations and high gene flow, is that underestimation of genetic differentiation due to the confounding allele size homoplasy is quite common [62]. On the other hand, the SNP discrimination power in ABFT has been able to detect population structure at a high hierarchical level, distinguishing populations from the Mediterranean Sea and the North Atlantic, but not from western Mediterranean Sea and the Bay of Biscay (Atlantic Ocean) [53]. Genetically homogeneous populations occupying large scale geographically distinct areas such as oceans and the Mediterranean Sea have also been detected through SNPs, in other tunas, such as the albacore (*Thunnus alalunga* [53, 63]).

In marine populations it is quite common to have large effective population sizes and relatively high rates of gene flow, resulting in a great difficulty to assess population structure, due to low or no genetic differentiation between populations [60, 64]. There have been cases where genetic distinction among populations is concealed by high mutation rates and extreme marker heterozygosity that result in a signal of low differentiation, and careful thinking prior to the interpretation of levels of differentiation is imperative [65, 66]. Therefore, although biologically significant differences may exist, those are not always detected statistically [60]. It comes as no surprise that in such cases significant spatial patterning is uncovered when seascape features are used (e.g. [67]).

In respect to the model-based methods employed in this study, either their performance was hampered by violations in model assumptions (e.g., when running STRUCTURE with the microsatellite dataset) or provided clues of the existence of a single population. However, it has been documented that at levels of genetic differentiation similar to our study (<0.02) STRUCTURE models fail to perform [68]. The information content of a dataset has a significant influence on the performance of STRUCTURE [69], while incomplete lineage sorting could confound structure inference, particularly for weak population differentiation and regardless of the algorithm employed [70]. Furthermore, the inclusion of a large proportion of admixed individuals in a dataset requires a large number of loci for ancestry coefficients to be reliable [44]. In GENELAND analysis on the other hand, by assuming a pure origin of a sample from only one of the defined genetic clusters does not allow individuals of mixed ancestry [47], an assumption that may contradict the biological traits of ABFT.

The EEMS was not a good descriptor of the migration and diversity of ABFT in the Mediterranean Sea. This could be due to the sampling scheme used in this study or lack of signal in the data, leading the estimation of migration rates being driven only by the prior (i.e. no heterogeneity in migration rates), or due to the violation of the equilibrium in time assumption of the model or a combination of the above.

Given that EEMS analysis of the dataset of Riccioni et al. [22], with an extensive sampling scheme and a more informative content indicating population structure within the Mediterranean Sea, also proved to be a poor descriptor of migration and diversity, renders the violation of the equilibrium in time assumption as highly probable. The decline that ABFT stocks have experienced up to the late 2000s due to overfishing suggests a strong perturbation of the equilibrium that could take tens or hundreds of generations to be restored [60]. Another factor that should be taken into account is whether Euclidean or Great circle distances reflect the actual distance ABFT has to cover based on its biological requirements and, therefore, the suitable path from one locality to another (as in [71] and [72]).

Αlthough several types of investigations have improved our knowledge of ABFT life history and stock structure, significant gaps still exist and must be addressed to ensure sustainability of the species. The dynamic fluid medium of seas and oceans in combination with the species traits, necessitates novel approaches and methods of analysis that will bring together experts in genetics/-omics, ecology and oceanography.

The study of samples of a certain age class like the young of the year would undoubtedly assist the research of ABFT population dynamics. Collecting a larger sample size from each ‘population’, could probably ameliorate the bias of estimates of interest (e.g. F_ST_) in combination to the analysis of multiple independent genetic loci [60]. However, addressing low power by increasing locus sample size will not necessarily improve inference unless there is a change in analytical philosophy [73–75]. Knowing that genetic patterns are influenced by the synergistic interaction of both environmental factors and life history traits [5], it is anticipated that studies combining their effects may provide answers to difficult questions related to ABFT.

Analyzing ABFT genetic data under the discipline of seascape genetics is anticipated to unravel a different perspective of the species population structure where the relevant temporal scale will be determined by the spatial factor(s) of interest, the temporal stability of those spatial factors, and the dispersal behavior of the species [5]. It could provide the analytical framework under which major abiotic and biotic forces controlling ABFT recruitment could be identified, elucidating the spawning strategy of ABFT that is far more complicated than initially thought.

The degree of complexity of the ABFT population structure coupled with the potential impact of environmental changes on the spatial and temporal distribution of the spawning areas [11, 18, 23] render multiple and continuous fisheries monitoring a prerequisite in order to achieve an efficient and long-term ABFT conservation. As proposed by Cermeño et al. [17], combining genetics and archival tagging would be an important asset in resolving the population dynamics and migratory behavior that would benefit greatly if seen under the framework of seascape genetics/-omics.

## Conclusions

Concluding, unravelling the drivers of ABFT population diversity would require the consideration of important aspects of the species spawning behavior for the determination of the appropriate sampling design. Plasticity in the selection of spawning sites is influenced by the spatial and temporal variability in the location of major oceanographic features and environmental conditions, such as salinity and sea-surface temperatures [11, 76]. Since genetic differentiation and variability are highly depended on survival to reproductive maturity and not just dispersal, investigating factors influencing larval migration and survival will shed light to factors affecting dispersal. An important feature is that not all individuals present in the spawning grounds during the reproductive season are reproductively mature [23]. Furthermore, sampling design should account for the highly probable inclusion of samples in the wrong population due to the spawning fidelity displayed by ABFT, and the potential sampling of individuals before reaching and while migrating towards the respective spawning grounds [59]. The incorporation of such samples in a study will hamper the recognition of population structure rendering detection and elimination of mixed samples extremely important.

## Abbreviations

ABFT: Atlantic Bluefin tuna
ddRAD-seq: double digest restriction associated DNA sequencing
EEMS: estimation of effective migration surfaces
HW: Hardy–Weinberg equilibrium
IBD: isolation by distance
PCA: principal-component analysis
SNPs: single nucleotide polymorphisms
